# MetaProClust-MS1: an MS1 Profiling Approach for Large-Scale Microbiome Screening

**DOI:** 10.1128/msystems.00381-22

**Published:** 2022-08-11

**Authors:** Caitlin M. A. Simopoulos, Zhibin Ning, Leyuan Li, Mona M. Khamis, Xu Zhang, Mathieu Lavallée-Adam, Daniel Figeys

**Affiliations:** a Ottawa Institute of Systems Biology, University of Ottawa, Ottawa, Ontario, Canada; b School of Pharmaceutical Sciences, Faculty of Medicine, University of Ottawa, Ottawa, Ontario, Canada; c SIMM-University of Ottawa Joint Research Center in Systems and Personalized Pharmacology, University of Ottawa, Ottawa, Ontario, Canada; Chan Zuckerberg Biohub

**Keywords:** MS1-only, microbiome, metaproteomics, drug screening, bioinformatics, unsupervised learning, clustering, mass spectrometry, proteomics, machine learning

## Abstract

Metaproteomics is used to explore the functional dynamics of microbial communities. However, acquiring metaproteomic data by tandem mass spectrometry (MS/MS) is time-consuming and resource-intensive, and there is a demand for computational methods that can be used to reduce these resource requirements. We present MetaProClust-MS1, a computational framework for microbiome feature screening developed to prioritize samples for follow-up MS/MS. In this proof-of-concept study, we tested and compared MetaProClust-MS1 results on gut microbiome data, from fecal samples, acquired using short 15-min MS1-only chromatographic gradients and MS1 spectra from longer 60-min gradients to MS/MS-acquired data. We found that MetaProClust-MS1 identified robust gut microbiome responses caused by xenobiotics with significantly correlated cluster topologies of comparable data sets. We also used MetaProClust-MS1 to reanalyze data from both a clinical MS/MS diagnostic study of pediatric patients with inflammatory bowel disease and an experiment evaluating the therapeutic effects of a small molecule on the brain tissue of Alzheimer’s disease mouse models. MetaProClust-MS1 clusters could distinguish between inflammatory bowel disease diagnoses (ulcerative colitis and Crohn’s disease) using samples from mucosal luminal interface samples and identified hippocampal proteome shifts of Alzheimer’s disease mouse models after small-molecule treatment. Therefore, we demonstrate that MetaProClust-MS1 can screen both microbiomes and single-species proteomes using only MS1 profiles, and our results suggest that this approach may be generalizable to any proteomics experiment. MetaProClust-MS1 may be especially useful for large-scale metaproteomic screening for the prioritization of samples for further metaproteomic characterization, using MS/MS, for instance, in addition to being a promising novel approach for clinical diagnostic screening.

**IMPORTANCE** Growing evidence suggests that human gut microbiome composition and function are highly associated with health and disease. As such, high-throughput metaproteomic studies are becoming more common in gut microbiome research. However, using a conventional long liquid chromatography (LC)-MS/MS gradient metaproteomics approach as an initial screen in large-scale microbiome experiments can be slow and expensive. To combat this challenge, we introduce MetaProClust-MS1, a computational framework for microbiome screening using MS1-only profiles. In this proof-of-concept study, we show that MetaProClust-MS1 identifies clusters of gut microbiome treatments using MS1-only profiles similar to those identified using MS/MS. Our approach allows researchers to prioritize samples and treatments of interest for further metaproteomic analyses and may be generally applicable to any proteomic analysis. In particular, this approach may be especially useful for large-scale metaproteomic screening or in clinical settings where rapid diagnostic evidence is required.

## INTRODUCTION

Studying community dynamics using metaproteomics has recently become more common due to its ability to evaluate both the taxonomic and functional compositions of samples (1). In a recent perspective, Kleiner (2) describes the strengths of studying the metaproteome and the abundance of questions that can be answered only through metaproteomic technology. For example, through metaproteomics, we can understand the molecular and cellular phenotypes of microbial communities, the energy sources of individuals in a community (3), and the functional changes introduced through posttranslational modifications (4).

One area where metaproteomics is particularly useful is in the study of human gut microbiomes and their connections to human health and disease. There is growing evidence to suggest that gut microbiome compositional and functional dysbiosis is driving human disease, as observed for inflammatory bowel disease (IBD) (5, 6), asthma (7), multiple sclerosis (8), obesity (9), type II diabetes, and cardiovascular diseases (10). In addition, xenobiotics can cause significant impacts on gut microbiomes (11), warranting further investigation into how compounds may affect the gut microbiota. Recently, Li et al. (12) introduced Rapid Assay of Individual Microbiome (RapidAIM), an *in vitro* assay used to assess the microbiome’s response to drugs using high-throughput metaproteomics and metagenomics. Thus far, RapidAIM has been used to identify drugs that cause significant alterations to the gut microbiome (12), determine how structural analogs affect the gut microbiome (13), and explore how individual microbiome profiles vary in response to resistant starches (14).

Using metaproteomics as an initial screen for high-throughput studies of other experimental and clinical readouts, however, can be slow and expensive. For example, deep metaproteomics, which is used for microbial strain-level data resolution, requires upwards of 240-min liquid chromatography-tandem mass spectrometry (LC-MS/MS) gradients (15). In addition, microbiome data can be challenging to analyze due to the large amount of compositional variability observed between individuals (16). Stemming from taxonomic variability, the effects of xenobiotics on gut microbiomes also have large variations when tested on multiple microbiomes (12); thus, it is essential to identify robust effects when considering high-throughput studies.

Large interest in high-throughput microbiome experiments necessitates research into reducing data acquisition time requirements. Such a reduction would improve the ability to screen large numbers of samples rapidly. A strategy that can accomplish this reduction is MS2-independent proteomics, which is founded on peptide mass fingerprinting (17–20). Workflows for short-gradient MS1-only proteomics, like DirectMS1 (21), offer the ability to identify proteins from retention times (RTs) and mass-to-charge (*m/z*) ratios of MS1 features. However, DirectMS1 remains suitable for single-species samples and has been thoroughly tested only on single-cell-line samples. Unfortunately, microbiome metaproteomic samples are more complex due to the presence of multiple microbial species and, depending on the sample type, can contain eukaryotic host proteins that may be of interest. In addition, the common approach to metaproteomics, data-dependent acquisition (DDA), acquires MS2 spectra for peptide identification based on the MS1 precursor ion intensity. As such, novel computational approaches are required for data analysis of MS1-only metaproteomic data, particularly for large-scale high-throughput studies.

In this study, we introduce MetaProClust-MS1 (MPC-MS1), a bioinformatic tool developed for screening data from high-throughput microbiome studies by clustering experimental treatments and conditions. In brief, the MPC-MS1 framework uses independent-component analysis (ICA) matrix decomposition and k-medoid clustering to identify clusters of coexpressed MS1 features. An eigenfeature is then calculated for each cluster and is used to summarize the intensity patterns of the MS1 features in a cluster across all experimental conditions. The summarized intensity values are then correlated with microbiome treatments or conditions, where a correlation distance matrix is used for hierarchical clustering of microbiome conditions. Thus, high-dimensional and potentially noisy MS1 feature intensity data are reduced to summaries of microbiome treatments that can be used for a quick screen of treatment effects on the microbiome or for clinical diagnosis groupings.

As a proof of concept, we demonstrate the ability of MPC-MS1 using five different data sets and three unique experiments. The first experiment considered the interactions of xenobiotics and the gut microbiome. Data were acquired by MS1 only and a short 15-min chromatography gradient (data set 1) and by MS/MS with a longer 60-min gradient (data set 2). We also extracted only the MS1 features from the longer 60-min gradient (data set 3). In addition, we tested MPC-MS1 on a previously published clinical IBD metaproteomics data set (acquired by MS/MS) containing samples from 71 individuals (data set 4). Finally, we also tested the compatibility of MPC-MS1 on single-species proteomics data from the cerebellum tissue of Alzheimer’s disease (AD) mouse models that were also originally acquired by MS/MS (data set 5). We observed similar clusters identified by MPC-MS1 across MS1-only and MS/MS data, indicating that our proposed MPC-MS1 approach may offer an alternative to MS/MS bottom-up metaproteomics for screening microbiomes. In addition, reanalysis of the clinical IBD metaproteomics data set and the Alzheimer’s disease mouse model proteomics data set successfully separated disease samples. These results highlight the abilities of MPC-MS1 for clinical research and its general applicability to a wide range of data types, including the reanalysis of previously reported MS/MS data and single-species proteomics data, while considering only MS1 features. The discriminative ability of MPC-MS1 and the potential to prioritize samples and conditions of interest for further exploration by metaproteomics will greatly accelerate high-throughput metaproteomic studies while reducing resource requirements. Finally, the ability to identify robust individual-independent disease patterns may be particularly useful in clinical settings and showcases the potential general applicability of MPC-MS1 to any metaproteomics and proteomics data set.

## RESULTS

**FIG 1:**
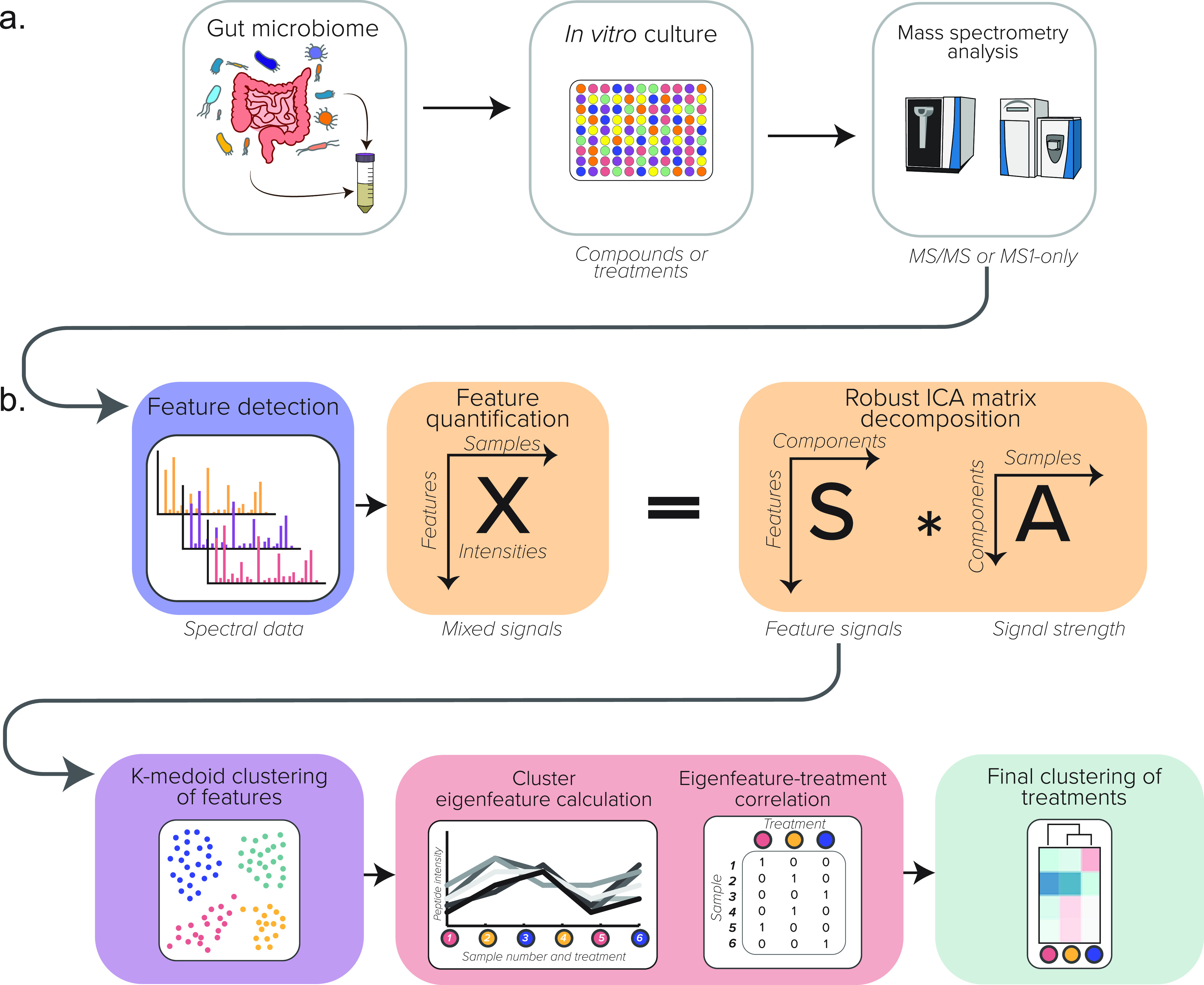
Illustrated schematic of the computational MPC-MS1 workflow. (a) Experimental workflow. (b) The MPC-MS1 framework, describing feature detection and quantification from MS1-only spectra, ICA matrix decomposition, k-medoid feature clustering, eigenfeature calculation and correlation with microbiome treatment, and the final clustering of microbiome treatments by hierarchical clustering.

MPC-MS1 is a bioinformatic strategy for screening large-scale microbiome experiments using MS1 profiles (Fig. 1). MPC-MS1 uses computationally aligned feature intensities acquired from MS1-only mass spectrometry without peptide or protein identification. To compare MS1-only results to MS/MS results, an MPC-MS1-like strategy was also adapted to handle the identified peptides and their intensities for direct use with MS/MS data.

After feature quantification, MPC-MS1 decomposes an intensity matrix (features by samples), matrix *X*, using a robust ICA implementation. Clusters of MS1 features are identified using k-medoid clustering from the *S* matrix that originally describes the feature contributions of the original *X* matrix projected onto *n* components. Eigenfeatures, vectors representing a summary of all features of a cluster, are calculated and correlated with microbiome treatments or conditions. A distance matrix is calculated from these treatment correlations and is used for a final bootstrapped hierarchical clustering of sample treatments. The treatment clusters can then be used to identify groups of treatments or conditions that cause large or small effects on the community of interest in order to prioritize samples for further conventional metaproteomic analysis.

As a proof of concept, we tested MPC-MS1 on five data sets. The first three data sets (data sets 1 to 3) were acquired from the same drug-treated gut microbiome sample where a single gut microbiome was treated with five drugs with a variety of known effects on a gut microbiome (12) at three concentrations (see Materials and Methods). We first compared MPC-MS1’s ability to cluster data acquired only at the MS1 level (data set 1) to its ability to cluster data acquired by MS/MS (data set 2). We then compared the MPC-MS1 results from the more traditional MS/MS approach (data set 2) to those from MS1-only scans extracted from MS/MS raw files (data set 3).

Data set 4 was used to test the ability of MPC-MS1 to discriminate between complex metaproteomic samples, in this case using MS1 features extracted from clinical mucosal luminal interface (MLI) aspirates from pediatric IBD patients. We then compared our MPC-MS1 results to those reported previously using conventional MS/MS metaproteomics.

Finally, we used our last data set (data set 5) to assess MPC-MS1 on single-species samples using hippocampus samples of AD mouse models. The original study evaluated the effects of a small molecule; therefore, we compared our results with those reported previously by Adler et al. (22).

### Data set 1: drug-microbiome interactions using MS1-only acquisition.

**FIG 2:**
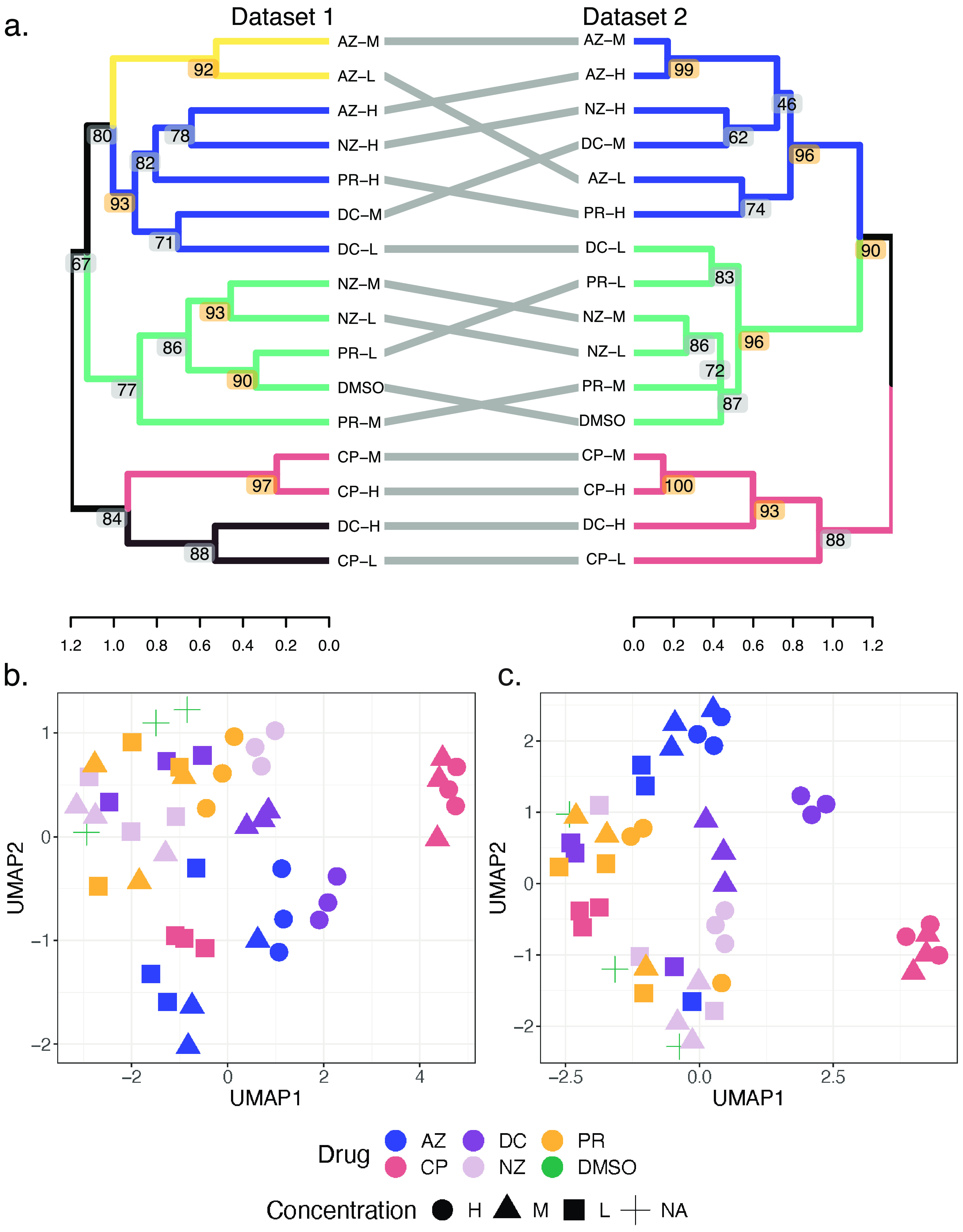
Drug-microbiome treatment clustering. (a) Hierarchical clustering of drug concentration treatments from data sets 1 and 2. Using silhouette scores, k values of 5 and 3 were chosen for dendrogram cutting and treatment clusters for data sets 1 and 2, respectively. Bootstrap AU *P* values are represented at each node, and significant AU values (AU *P* value of >0.9) are highlighted in orange. Dendrogram heights represent the average correlation distances between all intercluster pairs. (b) UMAP projections of log_2_ intensity values for data set 1. Drug treatments are represented by colors, with azathioprine (AZ) in blue, ciprofloxacin (CP) in pink, diclofenac (DC) in light purple, nizatidine (NZ) in dark purple, paracetamol (PR) in yellow, and DMSO (negative control) in green. Drug concentrations are represented by different shapes, where high (H) is indicated with circles, medium (M) is indicated with triangles, and low (L) is indicated with squares. A single concentration of DMSO was used and is represented as a cross. (c) UMAP projections of log_2_ fold change values for data set 2. Drugs and concentrations are represented as described above. NA, not applicable.

A total of 129,446 MS1 features were extracted using the OpenMS protocol as described in Materials and Methods. We retained 37,484 features (29%) after missing-value filtering. We then completed uniform manifold approximation and projection (UMAP) analysis on log_2_-transformed feature intensity data to first evaluate the discriminative ability of MS1-only features before ICA matrix decomposition using MPC-MS1. Clustering by drug concentration treatment was observed in visualized UMAP projections (Fig. 2b). Note that the samples from treatments with high (H) and medium (M) drug concentrations were typically farther from the control dimethyl sulfoxide (DMSO) samples than the samples from treatment with low (L) drug concentrations.

After data exploration by UMAP, principal-component analysis (PCA) was then used to determine the optimal number of components for the consequent ICA process (23) in MPC-MS1 as the number of appropriate ICA components is determined by each data set. In this case, 46 PCA components described at least 99% of the variation in the data. Thus, we completed 100 iterations of ICA matrix decomposition using 43 components. These calculations used 26.76 min of CPU time using 6 threads of a 2021 MacBookPro equipped with an Apple M1 Pro chip and 32 GB of memory.

The resulting *S* matrix described the contributions of features and was clustered using k-medoid clustering with k-medoids++ centroid initialization. MPC-MS1 automates k, or cluster number, choice by the maximum silhouette score, and in this case, a k value of 22 was selected.

Cluster labels were imported into R for eigenfeature calculation using log_2_ feature intensities. Correlations of cluster eigenfeatures with drug concentration treatment were calculated using Pearson’s correlation coefficient and used as a distance measure for drug concentration treatment clustering. Finally, silhouette score metrics identified five large robust drug concentration clusters (Fig. 2a). Bootstrapped hierarchical clustering also identified five smaller clades with approximately unbiased (AU) *P* values of >0.9 (Fig. 2a).

M and L concentrations of paracetamol (PR) and nizatidine (NZ) clustered with the control DMSO treatment, indicating that these drugs likely have a minimal impact on the gut microbiome (Fig. 2b). Notably, no high-concentration drugs clustered with the control treatment. Conversely, all concentrations of ciprofloxacin (CP) and high concentrations of diclofenac (DC) clustered the farthest from the DMSO control treatment (Fig. 2).

### Testing clustering with data censoring by MS1 feature intensity values.

Using our precomputed intensity quartiles, we tested if censoring data at intensity level thresholds would change the treatment cluster results. In other words, we filtered MS1-only features according to missing values identified by intensity quartiles to create two additional MS1-only data sets: “high-intensity” and “high- plus medium-intensity” feature data. We tested how correlated the clusters inferred from our full MS1 feature data set would be with the clusters identified by our censored data sets using cophenetic correlation. Neither the high-intensity nor the high- plus medium-intensity data set produced clusters that were significantly correlated with the full MS1 feature data set (*r *= 0.125 and 0.141, with *P* values of 0.148 and 0.105, respectively) (see Fig. S2 in the supplemental material). The high- and high- plus medium-intensity data sets were, however, significantly correlated with each other (*r *= 0.786; *P* < 0.0001).

10.1128/msystems.00381-22.2 FIG S2 Tanglegrams of clusters identified by data censoring. Tanglegrams were rotated to reduce entanglement measures using the step2side untangle function in the dendextend R package. (a) Final MS1-only clusters compared to high-intensity MS1 features. (b) Final MS1-only clusters compared to high- and medium-intensity features. (c) High-intensity clusters compared to high- and low-intensity features. Download FIG S2, PDF file, 0.5 MB. Copyright © 2022 Simopoulos et al. 2022 Simopoulos et al. https://creativecommons.org/licenses/by/4.0/ This content is distributed under the terms of the Creative Commons Attribution 4.0 International license.

### Data set 2: drug-microbiome interactions using MS/MS acquisition.

In total, 57,066 peptides were identified and quantified. After missing-value filtering, 29,470 peptides (51.6%) were retained for further analysis. We evaluated the discriminative abilities of the peptide log_2_-transformed intensity values identified by MS/MS using UMAP analysis (Fig. 2c). Similar to the MS1-only data in data set 1, drug concentration conditions typically clustered together. In addition, high drug concentrations are seen farther from the DMSO control samples.

For MPC-MS1, PCA was again used to determine the optimal number of components for the consequent ICA process (23), as the number of appropriate ICA components is determined by each data set. In this case, 46 PCA components described at least 99% of the variation in the data. Thus, we completed 100 iterations of ICA matrix decomposition using 46 components, which used 25.79 min of CPU time using 6 threads of a 2021 MacBookPro equipped with an Apple M1 Pro chip and 32 GB of memory.

Silhouette scores calculated from k-medoid clustering of the resulting *S* matrix suggested a k value of 48 to be the optimal number of peptide clusters. Peptide cluster labels were then imported into R for eigenfeature calculation and consequent correlation with drug concentration treatment. Using eigenfeature-treatment correlations, we clustered treatments using hierarchical clustering. Silhouette score evaluation identified three robust treatment clusters (Fig. 2a). Six nodes with significant AU *P* values indicate robust clustering of microbiome treatments.

Using data set 2, MPC-MS1 shows that all concentrations of CP and high (H) concentrations of DC cluster together and are the least similar to the DMSO control treatment (Fig. 2a). This clustering indicates that ciprofloxacin may have large effects on a gut microbiome even at a very low concentration, which aligns with its therapeutic indication as an antibacterial fluoroquinolone. Conversely, low (L) concentrations of DC and PR and L and medium (M) concentrations of NZ cluster with the control treatment.

We then compared clusters inferred from MS1-only feature intensities (data set 1) to peptides identified by canonical MS/MS (data set 2) (Fig. 2a). The overall topologies of both MS1-only- and MS/MS-inferred clusters remained relatively similar, and only one drug concentration treatment (DC-L) did not cluster consistently between the two data sets. We confirmed cluster visual similarity by cophenetic correlation calculation of the two dendrograms and found the clusters to be significantly similar and with more correlation than the censored data comparisons (*r *= 0.736; permutation *P* value of <0.0001) (Fig. S3).

10.1128/msystems.00381-22.3 FIG S3 Distribution of permuted cophenetic correlation coefficients comparing the clustering results of data sets 1 and 2. Permuted cophenetic correlation coefficients were computed for 10,000 iterations by dendrogram label swapping. The permutation *P* value of <0.0001 was calculated by the total number of absolute values of permuted correlation coefficients that were higher than the calculated cophenetic coefficient (*r *= 0.736). Download FIG S3, PDF file, 0.01 MB. Copyright © 2022 Simopoulos et al. 2022 Simopoulos et al. https://creativecommons.org/licenses/by/4.0/ This content is distributed under the terms of the Creative Commons Attribution 4.0 International license.

### Data set 3: drug-microbiome interactions using MS1 features extracted from MS/MS-acquired data.

We tested the ability of MPC-MS1 to reanalyze previously acquired MS/MS data by analyzing only the MS1 scans that can be extracted from an LC-MS/MS run. Of the 147,152 total MS1 features quantified, 32,833 features were retained after filtering (22.3%). First, using log_2_-transformed MS1 feature intensities extracted from the longer MS/MS gradient, we were still able to see clustering of drug concentration treatments from the UMAP analysis (Fig. S4a), similar to data sets 1 and 2.

10.1128/msystems.00381-22.4 FIG S4 Drug-microbiome treatment clustering. (a) UMAP projections of log_2_ intensity values of data set 3. Drug treatments are represented by colors, with azathioprine (AZ) in blue, ciprofloxacin (CP) in pink, diclofenac (DC) in light purple, nizatidine (NZ) in dark purple, paracetamol (PR) in yellow, and DMSO in green. Drug concentrations are represented by different shapes, where high (H) is indicated with circles, medium (M) is indicated with triangles, and low (L) is indicated with squares. DMSO did not follow the H, M, and L concentration schema and is represented by a cross. (b) Hierarchical clustering of drug concentration treatments from data sets 2 and 3. Using silhouette scores, k values of 3 and 4 were chosen for dendrogram cutting and treatment clusters for data sets 2 and 3, respectively. Bootstrap AU *P* values are represented at each node, and significant AU values (AU *P* value of >0.9) are highlighted in orange. (c) Hierarchical clustering of drug concentration treatments from data sets 1 and 3. Using silhouette scores, k values of 5 and 4 were chosen for dendrogram cutting and treatment clusters for data sets 2 and 3, respectively. Bootstrap AU *P* values are represented at each node, and significant AU values (AU *P* value of >0.9) are highlighted in orange. Download FIG S4, PDF file, 0.5 MB. Copyright © 2022 Simopoulos et al. 2022 Simopoulos et al. https://creativecommons.org/licenses/by/4.0/ This content is distributed under the terms of the Creative Commons Attribution 4.0 International license.

We then input the log_2_-transformed intensity values into the MPC-MS1 framework. Using PCA guidance, 43 components were considered for ICA matrix decomposition, which used 26.01 min of CPU time using 6 threads of a 2021 MacBookPro equipped with an Apple M1 Pro chip and 32 GB of memory. Silhouette scores computed from multiple iterations of k-medoid clustering found a k value of 22 to be the optimal k choice for MS1 feature intensity clustering. Again, silhouette scores were used to evaluate the optimal number of drug treatment clusters (Fig. S4b and c). In this case, four robust clusters (k = 4) were identified by the silhouette metric, while bootstrap analysis found three dendrogram nodes with significant AU *P* values.

We compared data set 1 (MS1 features extracted from a shorter MS1-only gradient) and data set 2 (MS/MS peptide matches from the same raw files) with data set 3. Neither comparison yielded significantly correlated dendrograms (Fig. S4b and c). However, note that CP-M, CP-H, and DC-H consistently clustered together in all three data sets.

### Data set 4: IBD data using MS1 features extracted from MS/MS data.

We then tested the MPC-MS1’s compatibility with a typical clinical metaproteomics data set considering only MS1 data. In this case, we also tested the ability of MPC-MS1 to distinguish between IBD diagnoses in uncultured microbiome samples from multiple individuals (*n* = 71).

These samples were run on the mass spectrometer over a span of a year from 21 June 2016 to 3 June 2017. When considering all samples together, we observed clustering of samples corresponding to the MS run date (Fig. S6b), suggesting that MS1 features are more sensitive to small environmental changes than an MS/MS data-dependent acquisition (DDA) approach. To circumvent this challenge and to maintain similarity to the previous MS/MS analysis, we completed MPC-MS1 (from MS1 feature alignment to treatment clustering) on samples from each intestinal location separately.

10.1128/msystems.00381-22.5 FIG S5 Distribution of permuted cophenetic correlation coefficients comparing the clustering results of data sets 2 and 3. Permuted cophenetic correlation coefficients were computed for 10,000 iterations by dendrogram label swapping. The permutation *P* value of 0.2379 was calculated by the total number of absolute values of permuted correlation coefficients that were higher than the calculated cophenetic coefficient (*r *= 0.103). Download FIG S5, PDF file, 0.01 MB. Copyright © 2022 Simopoulos et al. 2022 Simopoulos et al. https://creativecommons.org/licenses/by/4.0/ This content is distributed under the terms of the Creative Commons Attribution 4.0 International license.

10.1128/msystems.00381-22.6 FIG S6 UMAP projections of all IBD samples calculated from MS1 feature intensities. (a) Samples colored by diagnosis, where control data points are drawn in light blue, UC data are in red, and CD data are in royal blue. Point shapes represent intestinal sites of each sample, where AC is indicated by circles, DeC is indicated by triangles, and TI is indicated by squares. (b) Samples are colored as a gradient by MS run date, where more recent samples are in purple and earlier samples are in yellow. Patient diagnosis is also represented as shapes, where control samples are circles, UC samples are triangles, and CD samples are squares. Download FIG S6, PDF file, 0.4 MB. Copyright © 2022 Simopoulos et al. 2022 Simopoulos et al. https://creativecommons.org/licenses/by/4.0/ This content is distributed under the terms of the Creative Commons Attribution 4.0 International license.

**FIG 3:**
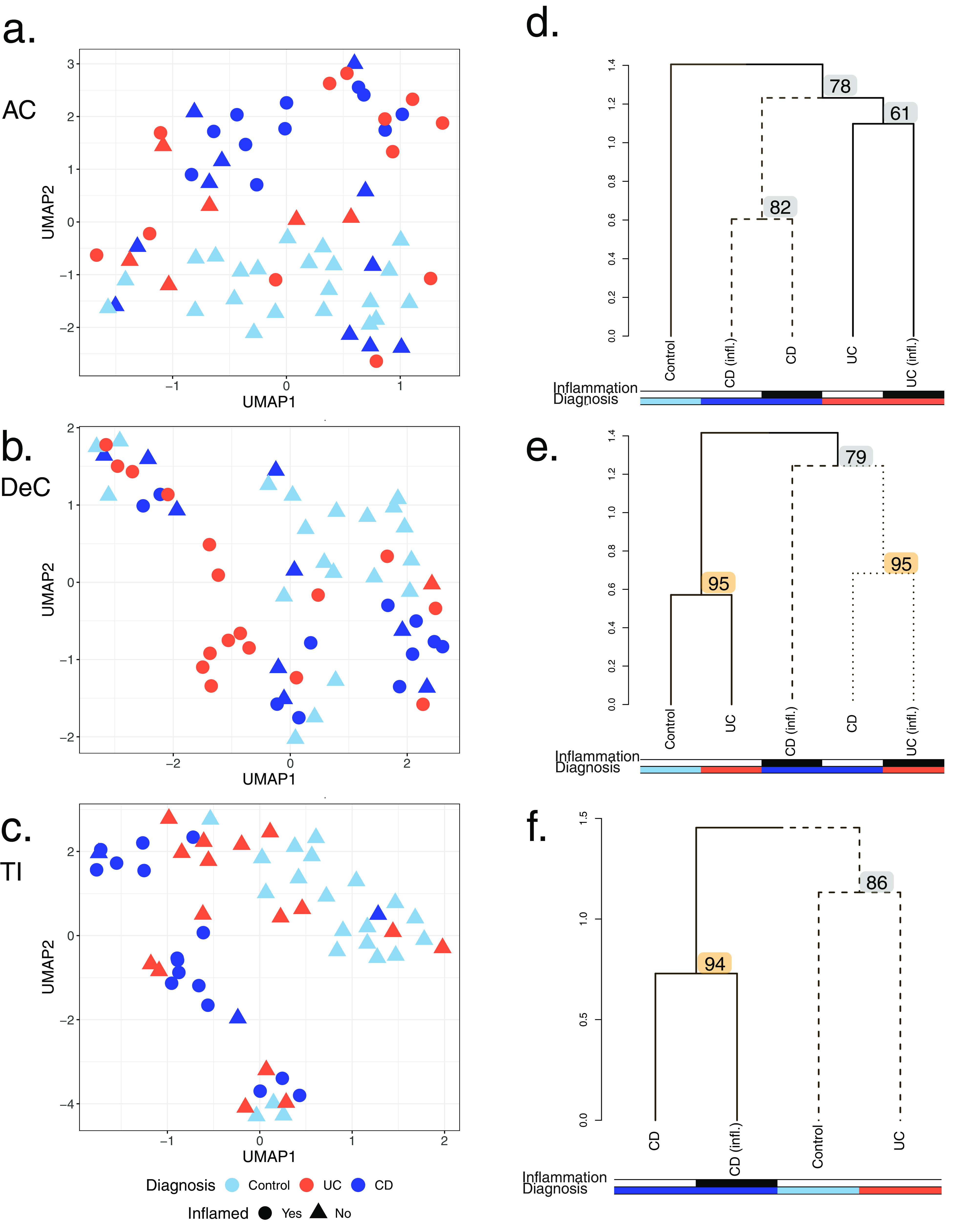
MS1 feature clustering of site-specific MLI aspirate samples from pediatric IBD patients. (a to c) UMAP projections of log_2_ MS1 intensity values. IBD diagnoses of UC and CD are represented in red and blue, respectively. Control patients are represented in light blue. Samples from inflamed sites are indicated by circles, while those from noninflamed sites are indicated by triangles. Each MLI aspirate site is represented separately: ascending colon (AC) (a), descending colon (DeC) (b), and terminal ileum (TI) (c). (d to f) Dendrograms inferred from MPC-MS1 analysis for each MLI aspirate site. Separate diagnosis clusters are represented by solid or dashed lines. The dendrogram height represents the average correlation distance of each intercluster pair. Bootstrap AU *P* values are represented at each node, and significant AU values (AU *P* value of >0.9) are highlighted in orange. Colored rows under each dendrogram represent inflammation status (top row) and patient diagnosis (bottom row), where UC and CD are represented in red and blue, respectively, and control samples are shown in light blue. If inflammation was observed, the colored bar is shaded in black. The sites are as follows: AC (d), DeC (e), and TI (f).

UMAP analysis demonstrated that, in general, MS1 feature intensities were sufficient to separate control samples from ulcerative colitis (UC) and Crohn’s disease (CD) samples (Fig. 3a to c). From this preliminary step, we continued MPC-MS1 analysis and clustered MS1 features into 10, 10, and 16 clusters of ascending colon (AC), descending colon (DeC), and terminal ileum (TI) samples, respectively, where ICA took 48.46, 23.38, and 23.19 min of CPU time for each intestinal site. Samples from each intestinal site were clustered individually, with k values of 2, 3, and 4 being chosen for AC, DeC, and TI using silhouette score metrics. Using bootstrap analysis, we identified robust diagnosis clusters for all three intestinal sites. Both AC and DeC analyses resulted in two of three nodes with AU bootstrap *P* values of >90 (Fig. 3d and e). The TI samples did not contain any samples from patients diagnosed with UC and inflammation, and thus, there were only two nodes available for bootstrap analysis, one of which resulted in an AU bootstrap *P* value of >90 (Fig. 3f).

### MPC-MS1-guided feature selection and generalized linear model development.

We then tested the putative ability to use MPC-MS1 feature clusters to guide biomarker discovery by selecting clusters highly correlated with each diagnosis at each intestinal site. We used MS1 features found in clusters with the maximum absolute correlation coefficient with the diagnosis of interest. Using the features in the selected cluster, we trained a logistic regression generalized linear model (GLM) classifier to predict the diagnosis of interest from all other diagnoses, while Lasso regularization was used for generalization and feature selection. A λ value was selected for each model separately using leave-one-out cross-validation. We continued with a model only if more than one MS1 feature remained after feature selection.

In total, six models met our criteria (Fig. S7 and Table S1). Three of these six models could predict an IBD diagnosis, while the other three could only distinguish between control and IBD patients. Five out of the six models had area under the curve (AUC) values of >0.71, as computed by leave-one-out cross-validation, suggesting that MS1 feature panels of between 3 and 10 features may be able to distinguish between different IBD diagnoses. The retention times (RTs) and mass-to-charge ratios (*m/z*) of all selected features were retained and are available in Table S1.

10.1128/msystems.00381-22.7 FIG S7 MS1 features and GLM metrics for IBD prediction models. The potential for MPC-MS1-guided biomarker discovery was explored by building binary GLMs with Lasso regularization to predict each IBD diagnosis at all three intestinal locations. Features were originally selected if they belonged to the cluster with the highest absolute correlation coefficient for the diagnosis of interest. Only models with more than one MS1 feature remaining after regularization were kept for further testing. Models at each intestinal site (AC, DeC, and TI) are visualized. The box plots show the mean log_2_ intensity values of cluster MS1 features that were plotted for each feature. Median values are described for each diagnosis. Lasso regularization was used for feature selection, and MS1 feature coefficients found to have a nonzero value are plotted in red, while all other features of the chosen cluster are plotted in gray. The resulting receiver operating characteristic (ROC) curve for each model using leave-one-out cross-validation is also shown. Download FIG S7, PDF file, 88.6 MB. Copyright © 2022 Simopoulos et al. 2022 Simopoulos et al. https://creativecommons.org/licenses/by/4.0/ This content is distributed under the terms of the Creative Commons Attribution 4.0 International license.

10.1128/msystems.00381-22.9 TABLE S1 Retention times and mass-to-charge ratios of MS1 features selected for all final GLMs for IBD prediction. Download Table S1, XLSX file, 0.01 MB. Copyright © 2022 Simopoulos et al. 2022 Simopoulos et al. https://creativecommons.org/licenses/by/4.0/ This content is distributed under the terms of the Creative Commons Attribution 4.0 International license.

### Data set 5: Alzheimer’s disease mouse model data using MS1 features extracted from data acquired by MS/MS.

Since MPC-MS1 was successfully applied to a controlled gut microbiome experiment (data sets 1 to 3) and a clinical gut microbiome experiment (data set 4), we were intrigued to expand the application of MPC-MS1 to a single-species proteomics experiment. This experiment considered the effects of a small molecule, a casein kinase 1δ/ε inhibitor, on the hippocampal tissue of AD mouse models (*n* = 23) with samples taken at two circadian time (CT) points.

To mirror the analysis described previously by Adler et al. (22), we analyzed both time points, circadian time 10 (CT10) and circadian time 14 (CT14), separately. We additionally analyzed all samples together to test if MPC-MS1 can offer novel insights into these data using only MS1 features. We found that preliminary UMAP analysis could differentiate among three conditions: AD mice treated with the drug, AD mice treated with the vehicle, and nontransgenic (NTg) mice treated with the vehicle (Fig. S8a to c). Of interest, AD mice treated with the drug clustered more closely with NTg mice treated with the vehicle whether time point data were analyzed together or separately.

10.1128/msystems.00381-22.8 FIG S8 MS1 feature clustering of Alzheimer’s disease (AD) mouse models after small-molecule treatment with samples taken at 2 time points (CT10 and CT14). (a to c) UMAP projections of log_2_ MS1 intensity values. Triple-transgenic AD mice treated with the small molecule are represented in red. Triple-transgenic AD mice treated with the vehicle are represented in blue. Nontransgenic mice treated with the vehicle are represented in yellow. (a) UMAP clustering of all time points together. CT10 time points are represented by circles, and CT14 time points are represented by triangles. (b) UMAP clustering of samples taken at CT10. AD samples are shown as circles, while samples from nontransgenic mice are shown as triangles. (c) UMAP clustering of samples taken at CT14. AD samples are shown as circles, while samples from nontransgenic mice are shown as triangles. (d to f) Dendrograms inferred from MPC-MS1 analysis. Separate clusters are represented by solid or dashed lines. The dendrogram height represents the average correlation distance of each intercluster pair. Bootstrap AU *P* values are represented at each node, and significant AU values (AU *P* value of >0.9) are highlighted in orange. Colored rows under each dendrogram represent the mouse type and treatment (as well as the time point when the sample was taken in panel d), where triple-transgenic AD mice treated with the small molecule are represented in red, triple-transgenic AD mice treated with the vehicle are represented in blue, and nontransgenic mice treated with the vehicle are represented in yellow. (d) Clustering results for combined time point analysis, including an additional bar below the dendrogram that is shaded in black if the sample was taken at CT10. (e) The MPC-MS1 clustering results for samples taken at CT10. (f) MPC-MS1 clustering results for samples taken at CT14. Download FIG S8, PDF file, 0.5 MB. Copyright © 2022 Simopoulos et al. 2022 Simopoulos et al. https://creativecommons.org/licenses/by/4.0/ This content is distributed under the terms of the Creative Commons Attribution 4.0 International license.

After the preliminary UMAP analysis confirmed that MS1 features can discriminate between mouse conditions, we continued the analysis according to the MPC-MS1 framework. PCA was used to select the ideal number of components for ICA. In this case, 22, 9, and 12 components were selected for data from both time points combined, CT10 data, and CT14 data, respectively. ICA matrix decomposition used 20.86 min of CPU time for both time points combined, 6.91 min for CT10 data, and 11.79 min for CT14 data. In all three data sets, a k value of 2 was found to be ideal (Fig. S8d to f). One node in the combined time point and CT14 dendrograms had bootstrap-computed AU *P* values of >90 (Fig. S8d and f).

## DISCUSSION

Although taxonomically and functionally informative, acquiring MS/MS metaproteomic data for microbiome research can be resource-intensive. In particular, data acquisition using the conventional tandem mass spectrometry approach is time-consuming, and this challenge is exacerbated in large-scale studies. In this work, we introduce MPC-MS1, a computational approach for microbiome screening that is free of MS2-dependent metaproteomic data requirements. Our approach can reduce the time and financial requirements that are typically required for large-scale studies by allowing treatment clustering before deep metaproteomics analysis. Using MPC-MS1 on our drug-microbiome interaction data sets, we were able to identify similar clusters using both MS1-only and MS/MS data sources, suggesting that MS1-only gradients can be useful for microbiome screening. On the other hand, our MPC-MS1 results from the IBD data set analysis suggest that MS1 features are able to discriminate between IBD diagnoses in a clinical setting, even using samples from many different individuals. By not relying on peptide identification from MS/MS or MS1 scans with high intensity chosen for MS/MS DDA, MPC-MS1 removes the restrictions imposed by choosing appropriate system-specific databases typically required for confident peptide spectral matches (PSMs) (24) and allows the algorithm to consider more analytes from which the data were acquired during MS1 scanning than an MS/MS approach. These benefits are exemplified by the wide range of data types that MPC-MS1 is able to analyze without specialized databases, including cultured microbiomes, clinical MLI samples, and mouse brain tissue.

### MPC-MS1 leverages the advantages of MS2-independent proteomics.

DDA, a common approach for MS/MS data acquisition in metaproteomics, can fall short in terms of the extent of information that can be extracted from such an experiment (25). For example, DDA selects only peptides with the highest intensities measured by MS1 for further fragmentation and eventual identification by MS2. Missing values can occur when a peptide is not selected for MS2 analysis, thus biasing MS/MS experiments toward higher-intensity peptides. As described previously by Ivanov et al. (21), there is developing interest in MS2-independent proteomics to remove the challenges of these technically imposed missing values and increase reproducibility in large-scale proteomics studies. Newer mass spectrometers have allowed DDA in MS/MS studies to provide deeper proteomic data. Although deeper analysis by MS/MS will increase the number of proteins identified and quantified, it remains insufficient to address the complexity of the gut metaproteome. Our results indicate that valuable information on the gut metaproteome is still lost in MS/MS experiments but can be retrieved from MS1 features. As such, not restricting feature identification by intensity in the MS1-only data sets (data sets 1, 3, and 4) may explain any inconsistencies in treatment clustering when we compared MS1-only clusters to those identified using MS/MS (Fig. 2; see also Fig. S4 in the supplemental material). These small inconsistencies in treatment clustering may mean that the features that are not selected for MS/MS and are quantified only by MS1 may play important roles in treatment separation.

Rechenberger et al. (26) recently described the challenges of clinical metaproteomic studies and highlighted that community-curated human gut microbiome databases, like the Integrated Human Gut Microbial Gene Catalog (IGC), can limit peptide identification. In the case described by Rechenberger et al. (26), over 50% of the peptides identified in their study were missing from the widely used database. Those authors instead suggest the use of a sample-specific database created by metagenomics. However, by being MS2 independent, MPC-MS1 removes the challenges of appropriate database selection or curation at the time of microbiome screening. In addition, MPC-MS1 offers unbiased insights into overall microbiota community profiles while eliminating the computational and financial requirements of metagenomic database construction and PSM identification for a preliminary screen. For example, our MPC-MS1 analysis using MS1-only features of the IBD data set (data set 4) could confidently separate control patients from those with IBD considering AU bootstrap *P* values of ≥78 and contained significant nodes separating IBD diagnoses with inflammation from those without inflammation. Conventional MS/MS metaproteomics microbiome analysis of these patients could only separate control patients from those with disease (6). These results show promise in using the MPC-MS1 approach in clinical settings where MS1-only diagnostics from complex microbiome samples may be useful. In addition, because MPC-MS1 is not limited by a database, our computational framework is generally applicable to any proteomics first-pass screen, from complex microbiome samples to single-cell proteomics. To demonstrate the capabilities of our computational framework, we tested MPC-MS1 on MS1-only data from an experiment that focused on the hippocampi of AD model mice treated with a small-molecule drug. MPC-MS1 successfully clustered these data when two circadian time points were analyzed separately, as completed in the original study, and identified that drug treatment shifts the hippocampal tissue of AD mice toward that of nontransgenic mice (Fig. S8). In addition, novel insights into these data were generated when MPC-MS1 also revealed that the hippocampi of AD mice cluster more closely with those of nontransgenic mice at CT14 than at CT10, suggesting that circadian time still has an effect on AD mice even after drug treatment.

While MS2-independent proteomic studies are of interest and seem promising, there are very few studies available that have used this approach. DirectMS1 is an example of a short-MS1-gradient tool for protein identification and quantification that was developed and tested on HeLa cells (21). Alternatively, RIPPER is an MS1-based proteomic and metabolomic tool that uses MS1 features without protein identification (27). RIPPER’s main goal is to use *t* tests to identify quantitative differences between two treatments. To our knowledge, MPC-MS1 is the first bioinformatic tool that considers MS1 profiling of metaproteomic data and the first MS1-only tool to accept more than two treatment conditions in its analysis. Because MPC-MS1 was developed with complex, multispecies samples in mind, MPC-MS1 is currently limited to treatment separation and does not identify peptides from MS1 features. However, in the future, MPC-MS1 can be extended for protein identification through MS1 features using peptide mass fingerprinting (17–20) and an MS1-only search workflow (28). Nonetheless, this type of approach remains to be tested on complex metaproteomic data and is currently beyond the scope of our study.

Methods other than MS2-independent proteomics have also been suggested for the reduction of resources required for proteomic studies. For example, Meyer et al. (29) maintained MS/MS and instead removed liquid chromatography (LC) to perform data-independent acquisition (DIA) proteomics by direct infusion with the use of high-field asymmetric-waveform ion mobility spectrometry (FAIMS). While FAIMS, and ion mobility in general, presents an intriguing avenue in proteomics, the complexity of microbiome metaproteome samples may challenge its utility. For instance, the compensation voltage must be either kept fixed or scanned across a narrow range to prevent the excessive prolongation of the duty cycle (30). Therefore, DIA via direct infusion and FAIMS may not be an appropriate approach for metaproteomic samples. However, in our proof-of-concept study, we demonstrate the success of not only rapid metaproteomic screening for microbiome treatments but also the possibility of using short MS1 gradients in microbiome studies. While we had originally tested data censoring of MS1 features due to the inherent inclusion of noise in MS1-only data, the reduction in the cluster similarity of the censored data to those inferred from the full-feature data set (data set 1) suggests that the so-called “noise” of MS1-only data could be essential for treatment separation.

### ICA as a tool for biologically relevant cluster analysis.

The framework for MPC-MS1 was inspired by gene expression network analysis tools commonly used in transcriptomics. A recent meta-analysis explored multiple module detection techniques in this type of network analysis and evaluated the performance of each algorithm by comparing detected to known modules (31). Matrix decomposition methods, such as ICA, consistently achieved high scores with multiple evaluation methods, and previous research used ICA to cluster bladder cancer subtypes (32) and to identify robust gene modules in Escherichia coli (23). ICA is often described as a “blind source separation” linear transformation method that is used to identify a linear representation of independent sources in a set of mixed signals (33). A common example using blind source separation is described as the “cocktail party problem,” where one attempts to determine what a person is saying in a noisy room, such as at a cocktail party (33). Similar to a “cocktail party,” one can use ICA to separate nonbiological or individualistic factors observed in microbiome data using this matrix decomposition method. MPC-MS1 uses ICA to identify robust modules of quantified features that are then used to cluster microbiome treatments.

### Potential use of MPC-MS1 to guide targeted proteomics.

The main intended use of MPC-MS1 is as a screening tool for more efficient, resource-conscious, and intentional metaproteomic research. In its current implementation, MPC-MS1 identifies feature clusters that summarize MS1 profiles to be used for clustering microbiomes by treatment. However, these MS1 feature clusters contain MS1 features with similar intensity profiles throughout an entire experiment that may warrant further investigation themselves or may be used for targeted metaproteomic analysis. For example, our IBD data set and corresponding MPC-MS1 results offer useful features for distinguishing IBD diagnoses (Fig. S7 and Table S1). While our current data set sample size may be too small for biomarker discovery, it is conceivable that with enough samples, an MS1 feature intensity cluster highly correlated with an IBD diagnosis may contain candidate biomarkers. Similarly, as described in this study, retention time and *m/z* information can be selected from feature clusters, which can then be used to focus research on specific features of interest for use in inclusion-list-based MS2 data acquisition.

### Future applications of MPC-MS1 for MS2-independent microbiome screens.

In our study, we outlined two case scenarios for the use of MPC-MS1 analysis where some ground truths are known (i.e., data sets 1 to 3, where high drug concentrations have a larger effect on the gut microbiome; data set 4, from endoscopic, histological, and radiological IBD diagnoses; and data set 5, from AD mouse models). Although we expect some gut microbiome differences between treatments, patients, or mice, the exact relationships between the samples are unknown. In the future, we believe that MPC-MS1 could be tested with synthetic gut microbiome communities to better understand the sensitivity of MPC-MS1 treatment clustering.

Ultimately, we hope that MPC-MS1 will be used in future applications where users are seeking to classify treatments with unknown effects on the microbiome. For example, in an expanded drug-microbiome interaction study, we may be interested in novel compounds that cluster with known microbiome-perturbing drugs. We could complete a drug-microbiome interaction experiment similar to the one in this study, with additional compounds with unknown effects on the microbiome. Compounds with interesting effects, such as nonantibiotic compounds clustering with antibiotics such as ciprofloxacin (CP), can then be selected for reanalysis by conventional metaproteomics to identify specific taxonomic and functional perturbations in the gut microbiome.

### Conclusion.

In conclusion, the MPC-MS1 computational pipeline is an effective bioinformatic tool for microbiome screening that can be used on any operating system that supports R and Python. MPC-MS1 is generally applicable to any proteomics experiment and can be used to reanalyze previously acquired MS/MS data. The applicability of using rapid MS1 profiles rather than time-consuming tandem mass spectrometry, even with complex microbiome data, means that MPC-MS1 can reduce the resources typically required for metaproteomics experiments. Importantly, our tool acts to complement large-scale RapidAIM assays and avoids the challenge of database selection and peptide identification because of its MS2-independent approach. By removing peptide identification, MPC-MS1 does not require a protein database or a false discovery rate (FDR)-reducing search strategy. Instead, MPC-MS1 is a rapid and unbiased tool that can be used to screen data from large-scale metaproteomic experiments and may be especially relevant for clinical studies. Finally, to our knowledge, MPC-MS1 is the first MS1 profiling tool specifically developed for the metaproteomic research community.

## MATERIALS AND METHODS

We tested MPC-MS1 on data from three separate experiments: a drug-microbiome interaction study, a study focusing on the gut microbiomes of pediatric IBD patients, and a study focused on the cerebellum tissue of Alzheimer’s disease (AD) mouse models. Three drug-microbiome interaction data sets were acquired from the same cultured samples where a gut microbiome was cultured for 48 h with either the control or drug treatment. The pediatric IBD data set considered mucosal luminal interface (MLI) aspirates obtained from 71 pediatric patients (<18 years of age) with suspected IBD (6). Finally, the AD data set looked at the therapeutic effects of a small molecule on triple-transgenic AD mouse models. Sample preparation and data acquisition methods are explained further below.

### Experiments, sample preparation, and data acquisition.

**(i) Drug-microbiome interactions.** Fecal samples were collected on-site into deoxygenated phosphate-buffered saline (PBS) (pH 7.6) with 10% (vol/vol) glycerol prereduced with 0.1% (wt/vol) l-cysteine hydrochloride. Within 10 min of collection, samples were weighed, transferred into an anaerobic workstation (5% H_2_, 5% CO_2_, and 90% N_2_ at 37°C), homogenized to 20% (wt/vol) in the same deoxygenated and prereduced buffer mixture, and filtered using sterile gauze to remove large particles to obtain the fecal inocula. Fecal inocula, as proxies for gut microbiomes, were stored at −80°C until they were used for RapidAIM. An *in vitro* RapidAIM assay (12) was used to assess the contributions of five drugs, azathioprine (AZ), ciprofloxacin (CP), diclofenac (DC), nizatidine (NZ), and paracetamol (PR), at three concentrations, high (H), medium (M), and low (L), to the human gut microbiome (feces/fecal) sample (Table 1). The H concentrations were previously identified by Li et al. (12) to have robust effects on a gut microbiome and were calculated considering the maximal oral dosage and average distribution in colon contents. Thus, H drug concentrations correspond to biologically relevant drug concentrations. Conversely, L and M drug concentrations of 100 μM and 500 μM were used to test the sensitivity of the MPC-MS1 framework. Dimethyl sulfoxide (DMSO) (5%, vol/vol) was used as a negative control and a drug solvent (12). Using RapidAIM, a gut microbiome (fecal sample) from a healthy individual was cultured for 48 h under anaerobic conditions with either the control treatment (DMSO) or drug treatment. Samples with control and drug treatments at each concentration level were cultured in replicates of 3. Proteins were extracted from cultured samples and digested with trypsin (Worthington Biochemical Corp., Lakewood, NJ). The research ethics board protocol (number 20160585-01H) for human stool sample collection was approved by the Ottawa Health Science Network Research Ethics Board at the Ottawa Hospital. Exclusion criteria for participation were an irritable bowel syndrome, inflammatory bowel disease, or diabetes diagnosis; antibiotic use or a gastroenteritis episode in the 3 months preceding collection; the use of pro/prebiotic, laxative, or antidiarrheal drugs in the last month preceding collection; or pregnancy.

**TABLE 1:** Drug concentrations used for RapidAIM treatment of gut microbiome (fecal) samples^*a*^

Drug or compound	Concn (μM)		
	L	M	H
AZ	100	500	2,700
CP	100	500	1,100
DC	100	500	3,100
NZ	100	500	4,500
PR	100	500	13,200

a Each drug was used at three different concentrations: low (L), medium (M), and high (H). High concentrations were those found previously by Li et al. to have an effect on the gut microbiome using RapidAIM (12). AZ, azathioprine; CP, ciprofloxacin; DC, diclofenac; NZ, nizatidine; PR, paracetamol.

### (a) MS1-only data acquisition.

The LC-mass spectrometry (MS) analysis was performed on a Q Exactive instrument (Thermo Fisher Scientific Inc., USA) with the Easy-nLC 1200 system (Thermo Fisher Scientific Inc., USA). All 48 samples were run in a randomized order. We loaded 1 μg of peptides on a high-performance liquid chromatography (HPLC) column packed in an electrospray ionization (ESI) spraying tip (75-μm inner diameter by 10 cm) packed with reverse-phase beads (3-μm/120-Å ReproSil-Pur C_18_ resin; Dr. Maisch GmbH, Ammerbuch, Germany) for separation. Formic acid (FA) (0.1%, vol/vol) in water was used as buffer A, and 0.1% FA in 80% acetonitrile was used as buffer B. The MS1-only method has full scans with a resolution of 7,000, a scan range from 200 to 1,400 *m/z*, an automatic gain control (AGC) target of 3E6, and a maximum injection time (IT) of 200ms. No dependent scans followed. A 15-min LC gradient was used: solvent B was linearly changed from 4% to 35% in 9 min, followed by another gradient from 35% to 80% in 2 min, and finally, the gradient was kept constant at 80% for 4 min. Example chromatograms are visualized in Fig. S1 in the supplemental material. Raw files were then used to create data set 1.

10.1128/msystems.00381-22.1 FIG S1 Example chromatograms for data set 1 (MS1 only) acquired on three different days. The top chromatogram in black is sample DC-H_1, the middle chromatogram in red is sample PR-M_2, and the bottom chromatogram in green is sample AZ-M_3. Download FIG S1, JPG file, 0.1 MB. Copyright © 2022 Simopoulos et al. 2022 Simopoulos et al. https://creativecommons.org/licenses/by/4.0/ This content is distributed under the terms of the Creative Commons Attribution 4.0 International license.

### (b) MS/MS data acquisition.

The same set of digests was analyzed by LC-MS/MS, and the analysis was performed using an UltiMate 3000 RSLCnano system (Thermo Fisher Scientific Inc., USA) coupled to an Orbitrap Exploris 480 mass spectrometer (Thermo Fisher Scientific Inc., USA) under similar chromatographic conditions on a tip column (75-μm inner diameter by 20 cm) packed with C_18_ reverse-phase beads (3-μm/120-Å ReproSil-Pur C_18_ resin; Dr. Maisch GmbH, Ammerbuch, Germany). A 60-min gradient of 5 to 35% (vol/vol) buffer B at a 300-L/min flow rate was used. The MS full scan was performed from 350 to 1,400 *m/z*, recorded in profile mode, and was followed by a data-dependent MS/MS scan of the 12 most intense ions, with a dynamic exclusion repeat count of 1 and an exclusion duration of 30 s. The resolutions for MS and MS/MS were 60,000 and 15,000, respectively. Raw files were then used to create data sets 2 and 3.

### Mucosal luminal interface aspirates from pediatric patients with IBD.

We used a clinical metaproteomic data set acquired from MLI aspirates from pediatric patients previously reported by Zhang et al. (6). Briefly, MLI aspirates from 71 patients were collected during diagnostic colonoscopy, during which patients either were diagnosed with Crohn’s disease (CD) or ulcerative colitis (UC), or were deemed to be non-IBD controls. Aspirates were collected from three different locations, the ascending colon (AC), descending colon (DeC), and/or terminal ileum (TI), to understand the biogeographical contributions of the gut microbiome and the host to IBD. MS/MS data acquisition was performed as described previously by Zhang et al. (6). Raw files under data set identifier PXD007819 were downloaded from the ProteomeXchange Consortium (34) via the PRIDE partner repository (35) and were used for data set 4.

### Therapeutic effects of a small molecule on Alzheimer’s disease mouse models.

We also tested MPC-MS1 on a single-species sample using data from an experiment that looked at the therapeutic effects of a small molecule on AD mouse models (22). Those authors tested the effects of PF-670462, a casein kinase 1δ/ε inhibitor, on triple-transgenic mouse AD models. AD models were treated with either the drug (AD_drug) or the vehicle (AD_vehicle) (20% [wt/vol] 2-hydroxypropyl-β-cyclodextrin) for 20 days. Nontransgenic (NTg) mice were given only the vehicle. Hippocampal tissue samples were taken at two circadian time (CT) points: CT10 and CT10. MS/MS data acquisition was performed as described previously by Adler et al. (22), and the raw files can be downloaded from the files under data set identifier PXD012281 from the ProteomeXchange Consortium (34) via the PRIDE partner repository (35). The raw files were used for data set 5.

### Data sets, feature identification, and data preprocessing.

**(i) Data set 1: drug-microbiome interactions with MS1-only acquisition.** MS1-level peptide features were first identified from the MS1-only data acquisition run of the drug-microbiome interaction experiment (see the section on drug-microbiome interactions above). We used the OpenMS suite of tools for MS data analysis (36); the exact commands and parameters used are available in the MPC-MS1 GitHub repository (https://github.com/northomics/MetaProClust-MS1/blob/main/bin/openMS_ms1_commands.sh). A brief overview of the OpenMS workflow is as follows. Thermo RAW files were first converted to .mzML format using MSConvertGUI from the ProteoWizard toolkit (37). Peak lists were identified using PeakPickerHiRes (using default parameters other than algorithm:signal_to_noise 0 and algorithm:ms_levels 1). Feature outputs as .XML files were identified from peaks using FeatureFinderCentroided. Retention times were aligned between MS runs using MapAlignerPoseClustering considering a maximum retention time difference of 300.0 s and a maximum *m/z* difference of 20.0 ppm. Finally, an experiment-specific consensus map was identified using FeatureLinkerUnlabeledQT and the same maximum retention time and *m/z* differences as the ones mentioned above. Linked features were then written to a .csv file using TextExporter for further analysis using the MPC-MS1 workflow.

MS1 feature intensities from the OpenMS workflow and experiment metadata were imported into R v4.0.4 (38). First, MS1 feature intensities were divided into quartiles. Features belonging to the lowest quartile (intensity below 5,858,867) were considered missing values for data filtering, and only features quantified in at least 50% of each drug treatment, including the DMSO control, were kept for further analysis. However, if a feature met our filtering criteria, we still considered intensities belonging to the lowest quartile for data analysis.

To compare the effects of using only high-abundance molecules, we then used the intensity quartiles computed as described above to calculate thresholds for two additional MS1-only data sets. Our first data-censoring data set, “high,” was created similarly to the complete MS1-only data set but instead used the lowest intensity of the highest quartile as the missing-value threshold (intensity of 29,190,200). Our second data-censoring data set, “high plus medium,” instead used the lowest value of the second highest quantile as the missing-value threshold (intensity of 12,626,200).

To prevent challenges with log_2_ transformation, we imputed missing data by k-nearest-neighbor imputation using the impute v1.64.0 R package (39) and default parameters other than colmax=0.95. Normalization followed missing-value imputation using the median ratio method in the DESeq2 v1.30.1 R package (40). Log_2_ transformation was completed on intensity values. We completed uniform manifold approximation and projection (UMAP) using the UMAP v0.2.7.0 R package (41) considering two components on log_2_-transformed intensity values to confirm the discriminative abilities of MS1-only-identified features using low-dimensional projection.

### (ii) Data set 2: drug-microbiome interactions with MS/MS acquisition.

Data set 2 considers the data acquired by MS/MS. The mass spectrum search and peptide quantification were completed using MetaLab 2.0 (4) and a closed-database search of the Integrated Human Gut Microbial Gene Catalog (IGC) (42) with a peptide FDR threshold of 0.01. Peptide-level intensities were used for further analyses to maintain similarity with MS1-only features.

Similar to data set 1, both experiment metadata and MS/MS-level data peptide intensities were imported into R v4.0.4 (38). Only peptides identified in at least 50% of each drug treatment, including the DMSO control, were kept for further analysis. Peptide intensities were normalized, missing values were imputed, and log_2_ fold change values were calculated as described above. UMAP was also completed as described above.

### (iii) Data set 3: drug-microbiome interactions with MS1 features extracted from MS/MS-acquired data.

We tested the ability to reanalyze MS/MS data by extracting MS1 scans from MS/MS drug-microbiome interaction experiment RAW files. First, Thermo RAW files were converted to .mzML format using MSConvertGUI from the ProteoWizard toolkit (37) with the conversion limited to the MS1 scan level. We identified peak lists and aligned the features limited to the MS1 scan level using OpenMS and the same commands as the ones described above for data set 1. MS1 features and metadata were again imported into R v4.04 (38). MS1 feature intensities were filtered by quartiles and normalized similarly to data set 1. Finally, samples were visualized using UMAP projections of MS1 intensities.

### (iv) Data set 4: IBD data with MS1 features extracted from MS/MS-acquired data.

We extracted MS1 features from a previously reported conventional metaproteomic experiment that measured microbe and host proteins from uncultured MLI samples from 71 individuals (6). We adjusted the OpenMS commands to identify peak lists and align MS1 features for a 4-h gradient. Specifically, we allowed a maximum retention time of 1,200 s and a maximum *m/z* difference of 50 ppm in both the MapAlignerPoseClustering and FeatureLinkerUnlabeledQT steps of the pipeline. Samples taken from each location (AC, DeC, or TI) were aligned and analyzed separately.

MS1 features and sample metadata for each location were separately imported into R v4.04 (38) and were filtered and normalized similarly to data sets 1 and 3. Samples were visualized by UMAP projections of their MS1 intensities.

### (v) Data set 5: Alzheimer’s disease mouse model.

We extracted MS1 features from a previously reported MS/MS experiment that looked at the effects of a small molecule on hippocampal tissues of AD mouse models compared to those of NTg mice (22). We first converted Thermo RAW files to .mzML format using MSConvertGUI from the ProteoWizard toolkit (37), limited to the MS1 scan level. Using the converted files, we identified peak lists and aligned MS1 features using OpenMS and the same commands as the ones described above for data set 1. MS1 features and metadata were again imported into R v4.04 (38). MS1 feature intensities were filtered by quartiles and normalized similarly to data set 1. Finally, samples were visualized using UMAP projections of MS1 intensities. We also completed UMAP for each time point (CT10 and CT14) separately.

### MetaProClust-MS1 implementation.

The MPC-MS1 framework was used to cluster microbiome treatments/diagnoses for all four data sets. The framework’s three main steps are outlined below (Fig. 1).

### (i) Independent-component analysis.

The Precision RNA-seq Expression Compendium for Independent Signal Exploration (PRECISE) ICA implementation was used for robust component calculation (23). Briefly, the PRECISE ICA implementation, originally compiled for transcriptome sequencing (RNA-Seq) analysis, completes multiple ICA calculations using random seeds. The source components (*S* matrix) identified from each ICA run are clustered, and the final robust components are described as the centroids from each identified cluster. The number of components is set by determining the number of principal-component analysis (PCA) components required to explain 99% of the variance. Using Python 3.7.5, we called the PRECISE run_ica.py script using default parameters, including 100 ICA iterations and a convergence tolerance of 10^−6^.

### (ii) k-medoid clustering.

We used k-medoid clustering to cluster the final *S* matrix using Python v3.7.5 and scikit-learn-extra v0.1.0b2. Clusters were computed from a correlation distance using the k-medoids++ initialization algorithm. We tested for cluster fit using k values of 10 to 50 and automated k choice by the maximum silhouette score. Depending on the data source from MS1 features or MS/MS-identified peptides, peptide or feature cluster labels were then exported for further analysis in R.

### (iii) Eigenfeature-condition correlation.

Using R, eigenfeatures were calculated for each peptide or feature cluster using singular-value decomposition (SVD) of each cluster’s intensity values. As described previously by Langfelder and Horvath (43), the first column of SVD is used as the eigenfeature vector for each cluster. These values can then be used to explore the relationships between clusters. We calculated Pearson’s correlation coefficients of eigenfeature values for each drug concentration treatment or IBD diagnosis. Relationships between peptide/feature clusters and drug concentration treatments were explored by hierarchical clustering. Hierarchical clustering was completed in R using Pearson’s correlation coefficient distance measure and the average linkage hierarchical clustering method. Robust clusters were identified by two independent methods, (i) silhouette score calculation and (ii) bootstrapping with 1,000 iterations, using the pvclust v2.2.0 R package (44), where clusters with approximately unbiased (AU) *P* values of >0.9 were considered robust.

### Comparing cluster dendrograms.

Drug concentration treatment clusters of the appropriate data sets were compared visually by tanglegrams and cophenetic correlation coefficient calculation. Cophenetic correlation was performed using the cor_cophenetic() function from the dendextend v1.15.1 R package (45). A permutation test using 10,000 iterations was used to estimate the statistical significance of the obtained cophenetic correlation values. To calculate significance by permutation, dendrogram labels of a data set’s acquired clusters were shuffled, and cophenetic correlation coefficients were calculated to measure similarity between another dendrogram and the permuted dendrogram. We counted the number of times that the permuted correlation coefficient was observed to be higher than the actual calculated value. Visual comparisons of clustering by tanglegrams were also performed using dendextend (45).

### MPC-MS1-guided feature selection and generalized linear model development.

We tested the ability of MPC-MS1-generated MS1 feature clusters to guide candidate biomarker discovery on data set 4. We used MS1 feature clusters inferred by MPC-MS1 to help guide feature selection by considering only features belonging to the cluster with the maximum absolute correlation coefficient for the diagnosis of interest. We estimated binary regression generalized linear model (GLM) classifiers on each diagnosis at each intestinal location using the glmnet R package (46). Leave-one-out cross-validation was used to choose the ideal value of λ where lambda.1se was selected for the final model to ensure that the most regularized model was used. Lasso regularization was also used for feature selection by setting the alpha value equal to 1. We considered only models where Lasso regularization selected more than one feature. The final model performance was again evaluated using leave-one-out cross-validation, and an area under the curve (AUC) score was calculated using the pROC R package (47).

### Data availability.

Raw files used to compile MS1-only data sets 1 to 3 were deposited at the ProteomeXchange Consortium (34) via the PRIDE (35) partner repository with the data set identifiers PXD024815 and PXD024845. The STORMS checklist can be found at https://github.com/northomics/MetaProClust-MS1/blob/main/data/STORMS_v1.03_simopoulos.xlsx (48). Modular and customizable code and example data sets are available on GitHub at https://github.com/northomics/MetaProClust-MS1. R notebooks describing the examples in this study and code for figure creation are also included.
