# Supplementary figures and images for: MetaProClust-MS1: an MS1 Profiling Approach for Large-Scale Microbiome Screening
Source: mSystems. 2022 Aug 11;7(4):e00381-22. doi: 10.1128/msystems.00381-22 (PMC9426440; doi:10.1128/msystems.00381-22)

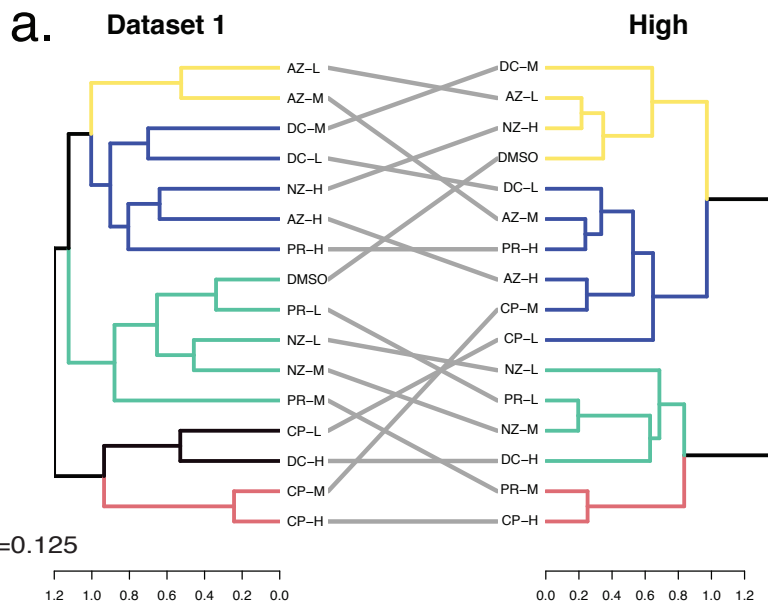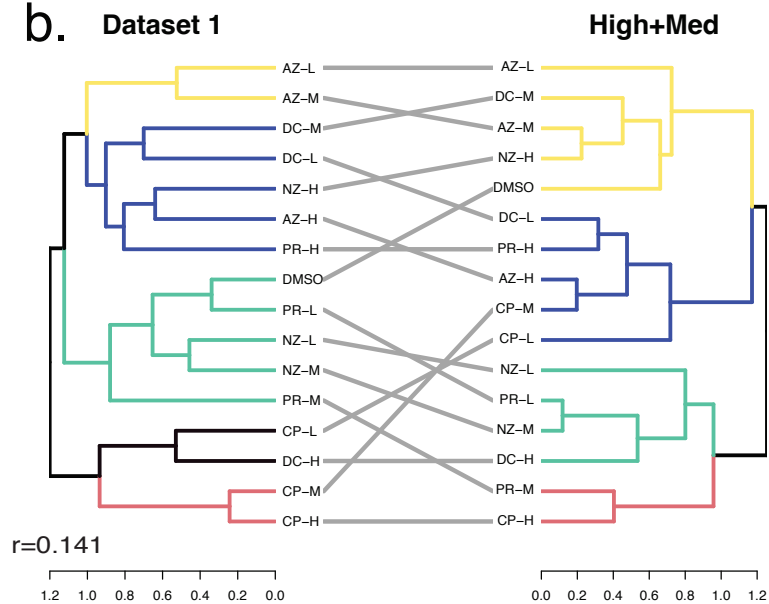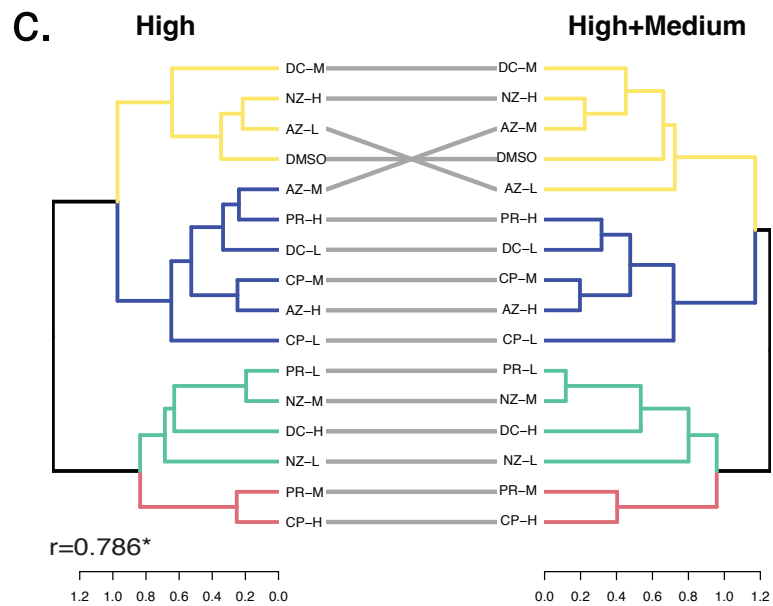

Supplement: FIG S2 [file msystems.00381-22-s0002.pdf]

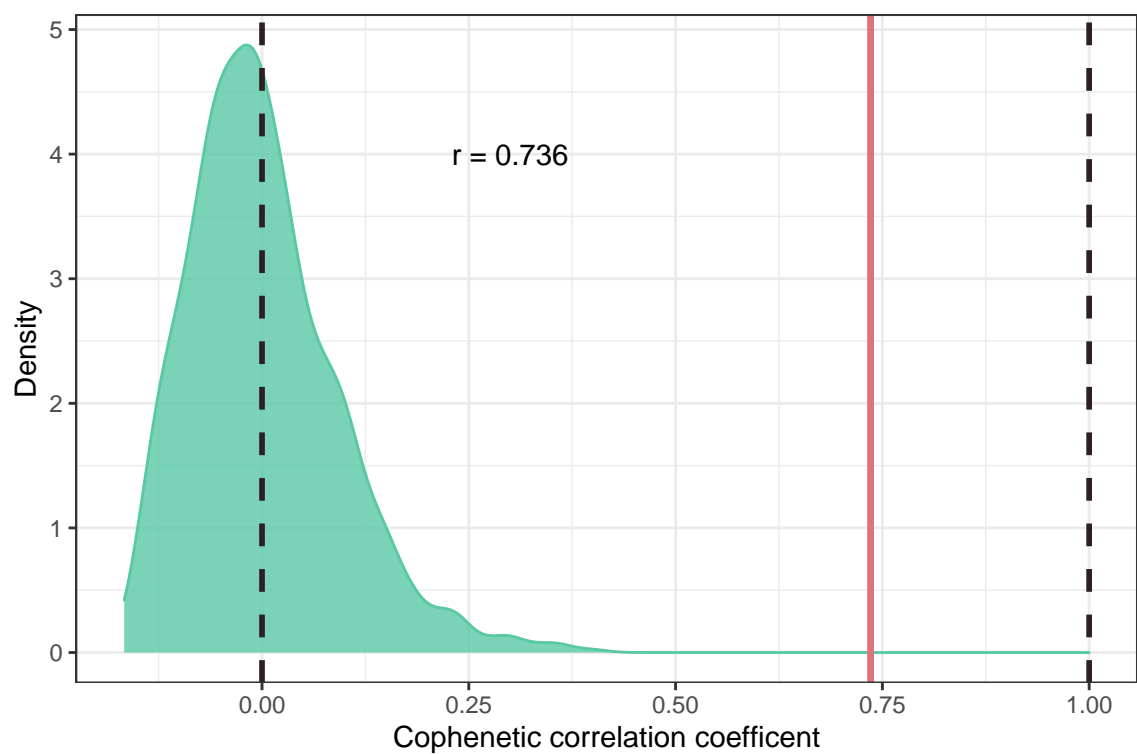

Supplement: FIG S3 [file msystems.00381-22-s0003.pdf]

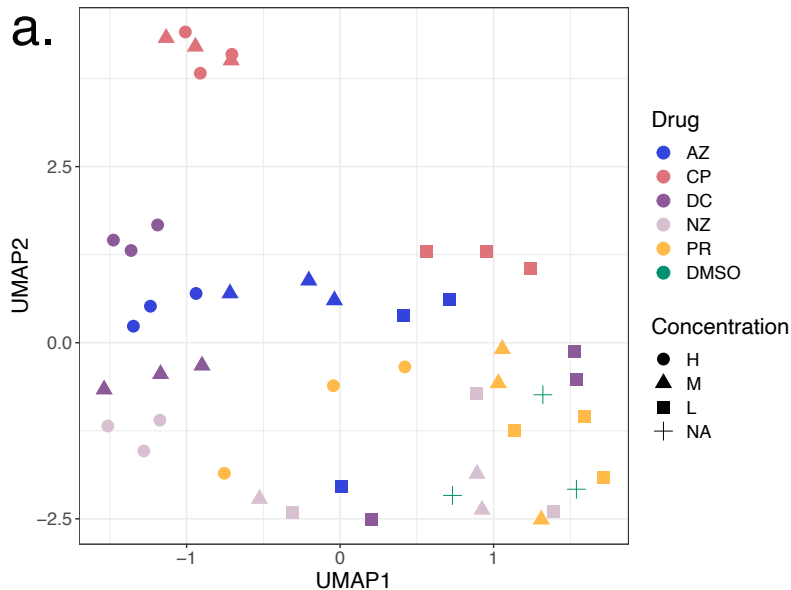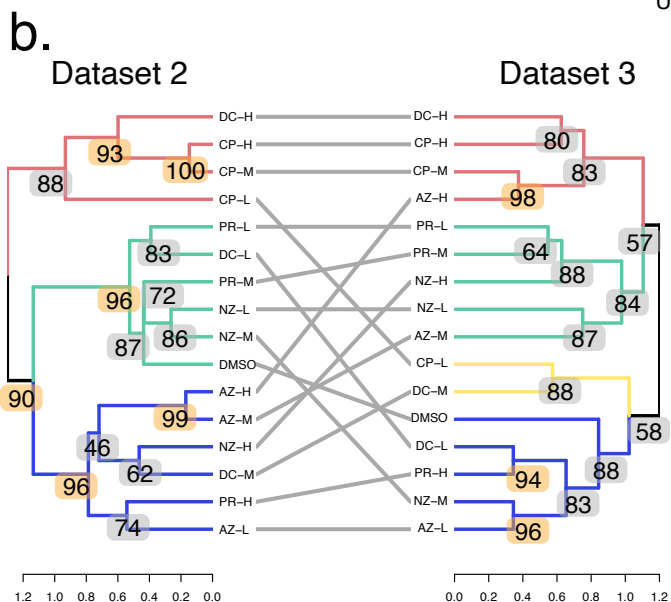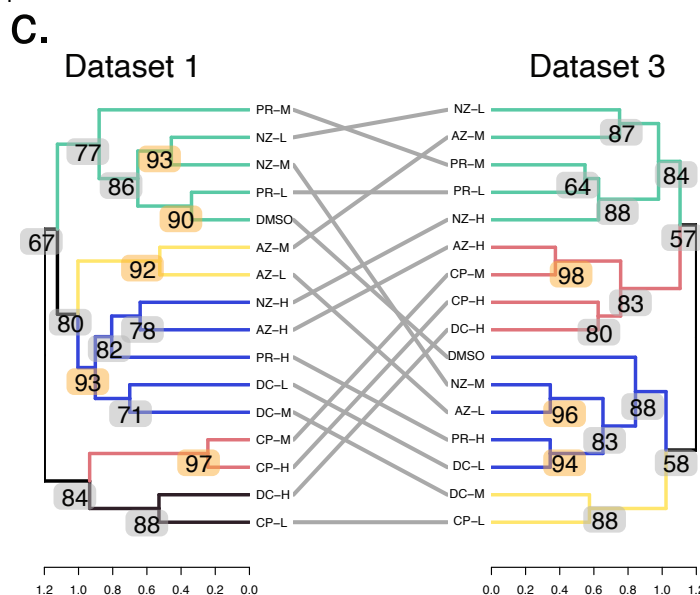

Supplement: FIG S4 [file msystems.00381-22-s0004.pdf]

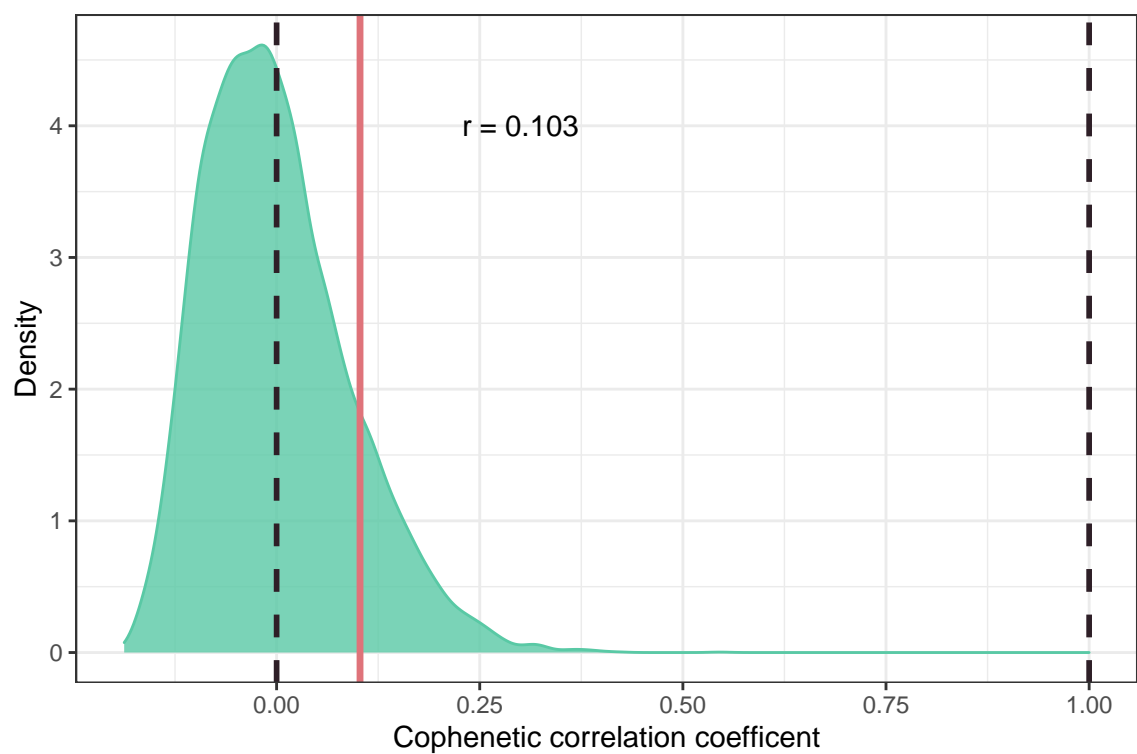

Supplement: FIG S5 [file msystems.00381-22-s0005.pdf]

a.

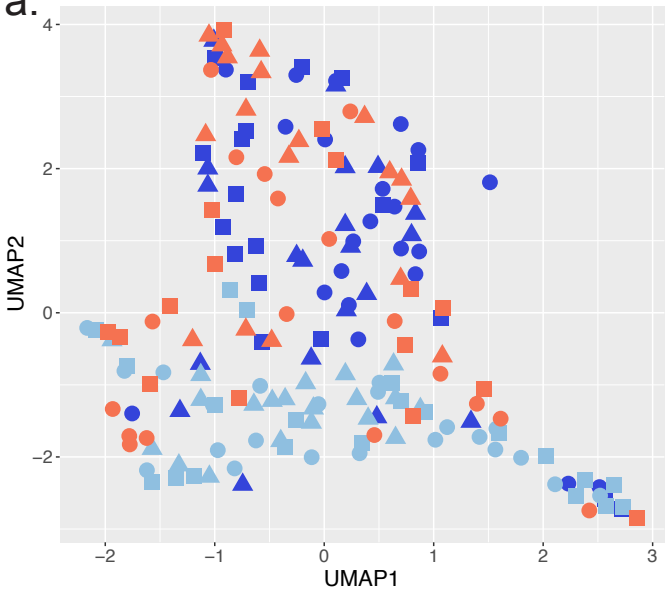

b.

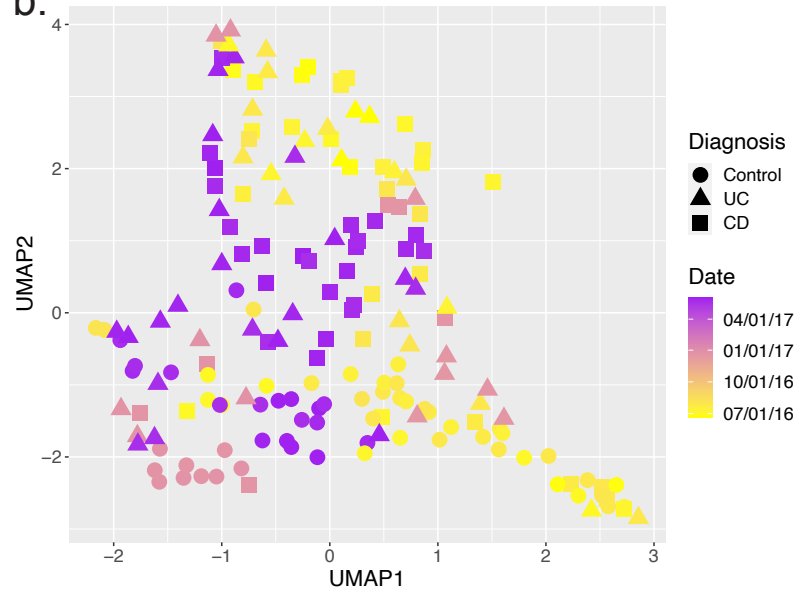

Supplement: FIG S6 [file msystems.00381-22-s0006.pdf]

AC

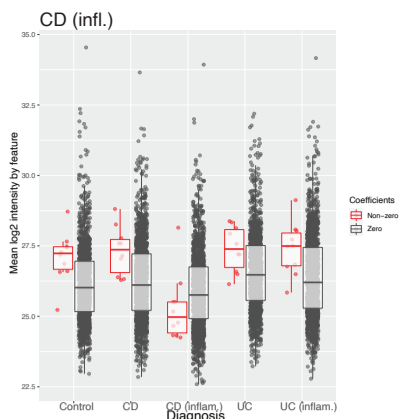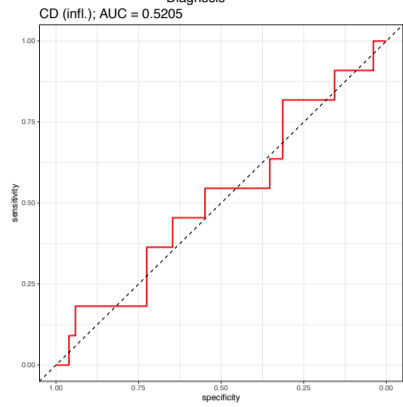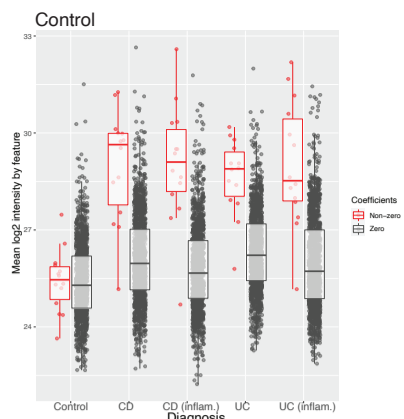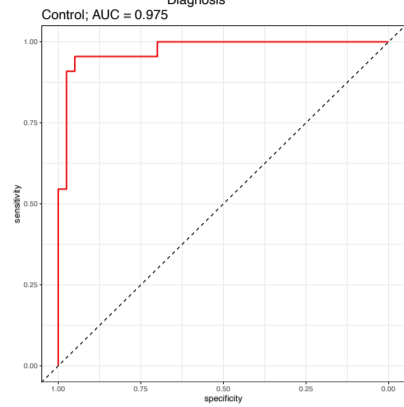

DeC

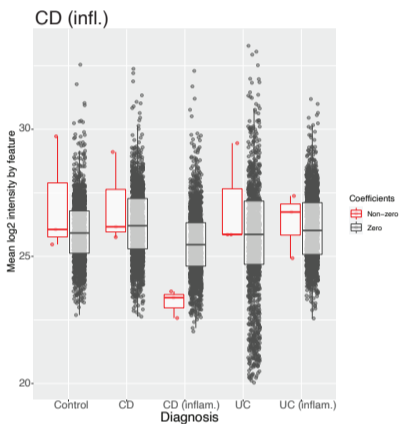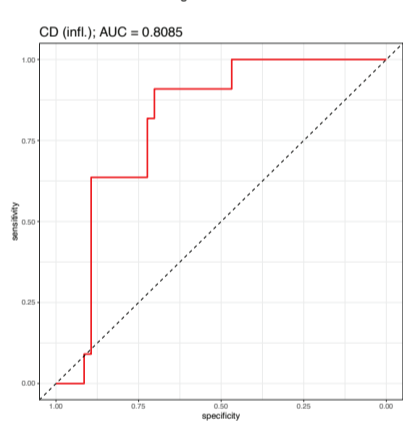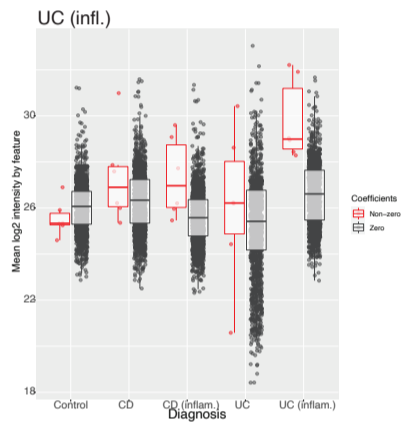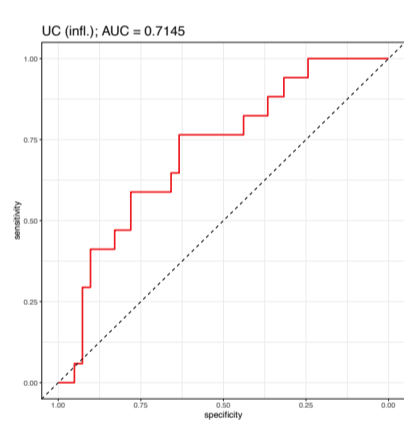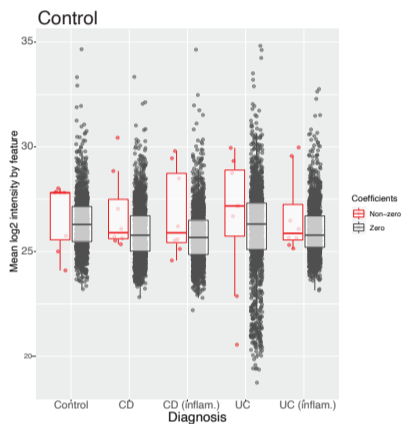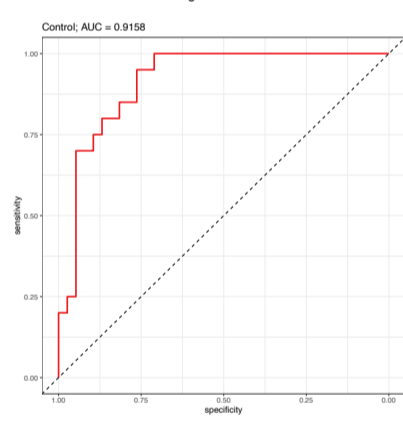

TI

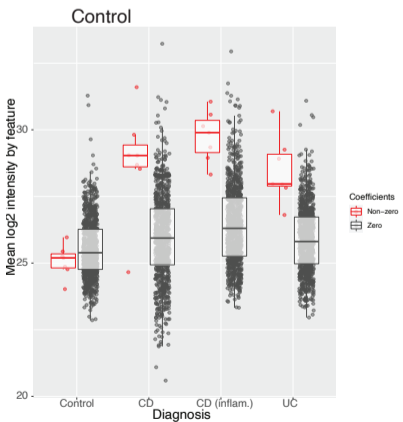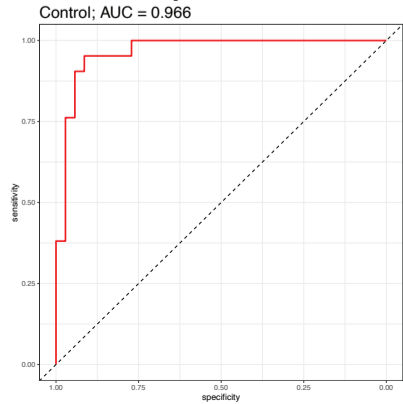

Supplement: FIG S7 [file msystems.00381-22-s0007.pdf]

CT10 &amp; CT14

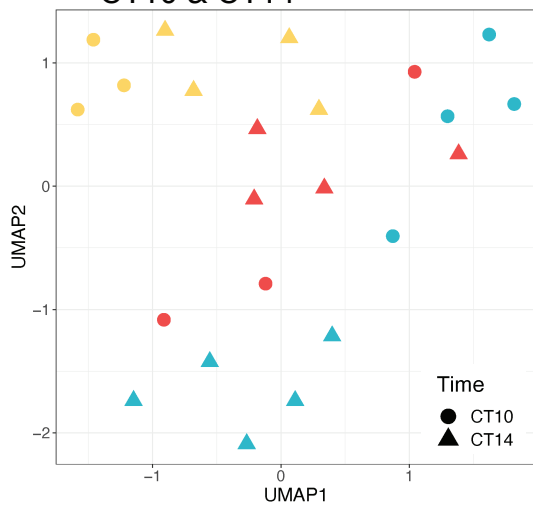

CT10

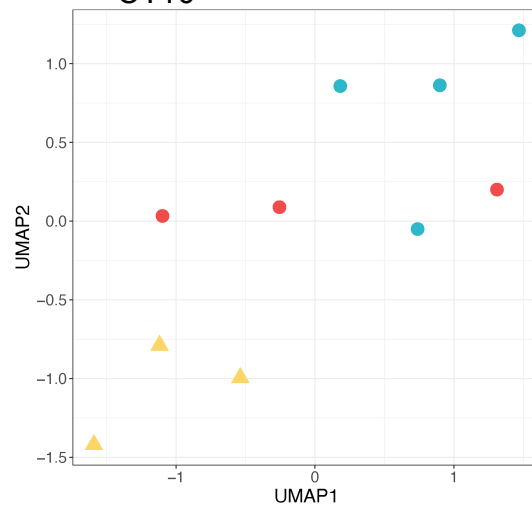

CT14

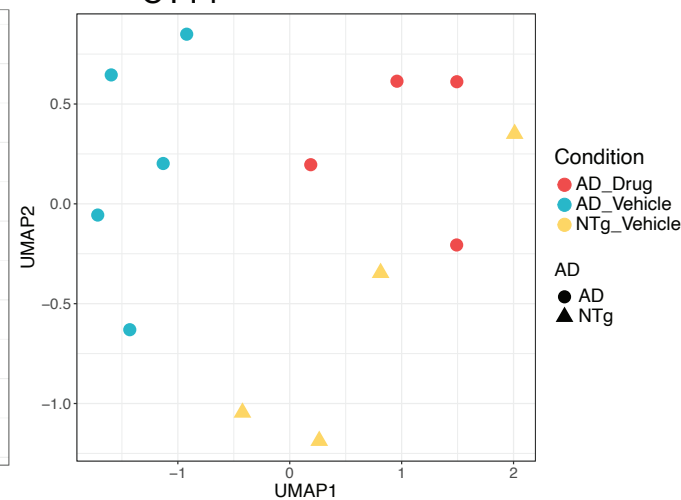

Condition  
 ● AD\_Drug  
 ● AD\_Vehicle  
 ● NTg\_Vehicle

AD  
 ● AD  
 ▲ NTg

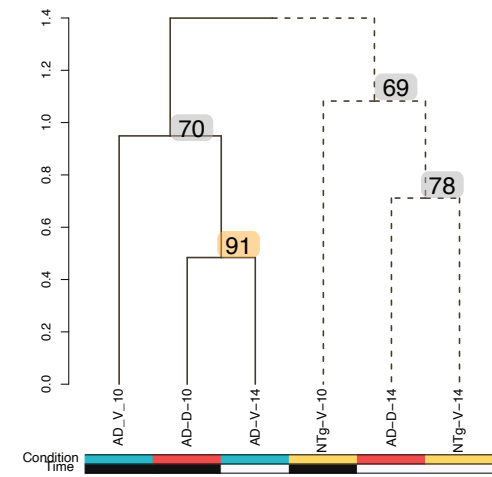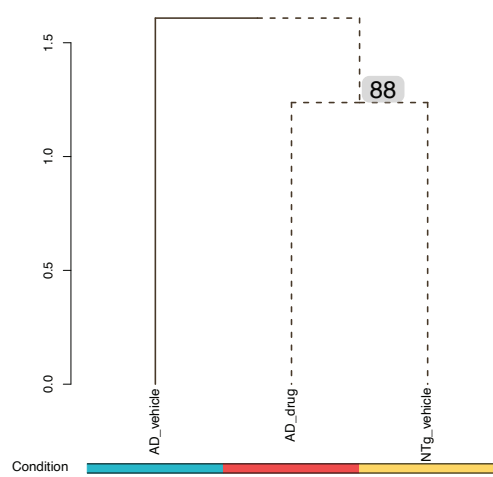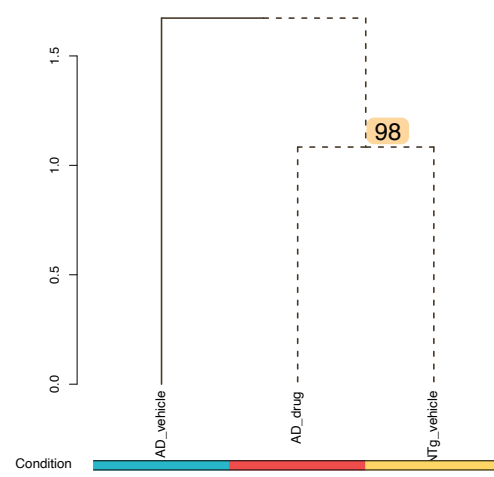

Supplement: FIG S8 [file msystems.00381-22-s0008.pdf]

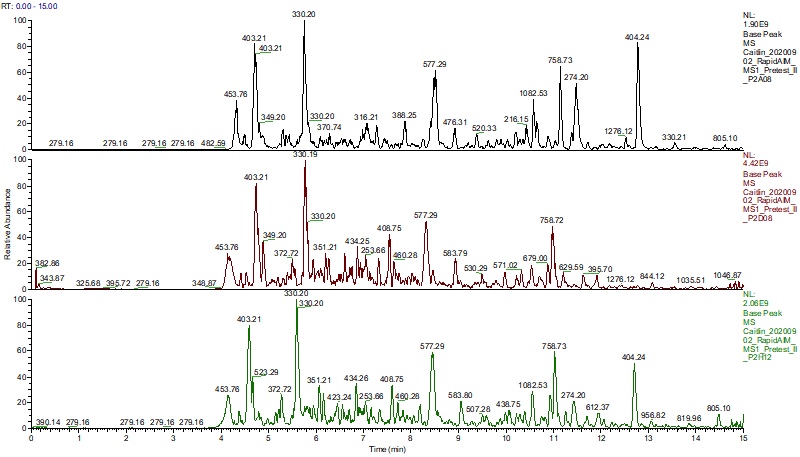

Supplement: FIG S1 [file msystems.00381-22-s0001.jpg]
